# An Essay Towards a Correct Theory of the Nervous System

**Published:** 1844-12

**Authors:** 


### REVIEWS.

---

Art. III.—*An Essay towards a Correct Theory of the Nervous System.* By John Harrison, M. D., Professor of Physiology and Pathology in the Medical College of Louisiana. Philadelphia: Lea & Blanchard. 1844. 8vo. pp. 292.

This work evinces a logical and acute mind. It abounds in discussions which require great closeness and patience of mental application. To do it justice in the way of a critical and analytical review, would require more space, and more time than we have at our disposal. This we regret, because we delayed the notice of the work thus long, in the hope that we should find leisure to go into a thorough examination of its doctrines. But what we are unable to do ourselves, we earnestly recommend our readers who have a taste for speculations in physiology to do. They will find, that Professor Harrison has displayed on all the topics discussed in his Essay an acumen and cultivation of intellect, which cannot fail to excite their admiration; and, in his ample store of facts collected from all quarters, they will be rewarded by a fund of valuable information, whatever may be their opinions as to the correctness of the author's conclusions. Upon these we do not feel prepared to pass our judgment. Some of them strike us, according to the light we now have, as somewhat ultra; still we will not undertake to say that future researches will not sustain them. The doctrine of the following passage, for example, is one which, we conjecture, few readers will be inclined implicitly to receive. Says the author—

"We insist, therefore, that it is altogether from misconception of the nature of chemical combinations, that it is so roundly asserted that matter operating by means of its own forces, cannot produce an organic compound. But why is it, then, that the chemist cannot in his laboratory produce an organic compound? Simply because he cannot place his materials under those circumstances *essential* to their production. Sugar and gum are composed of carbon and water; but regard, for an instance, the multiplicity of forces, conjoined and operating at the same moment, required to make water and carbon unite. In the first place the leaf must be of a certain chemical constitution, be possessed of a certain structure, and be well supplied with water. There must also exist a certain temperature; light must be present; and the leaf must be of a green color. If any one of these conditions be withdrawn, the union of the two substances will not take place. Now, will it be contended, that the chemical elements of the leaf being given, with the whole structure of the living plant, together with every other circumstance the same; the carbonic acid of the atmosphere would not be decomposed, and that the carbon would not unite with the water? That, in short, there must be introduced in the case the agency of an occult, mysterious principle, before the union could be effected? Assuredly those who thus argue, are under the *onus probandi;*—they must prove their assertion or their *vital principle* must remain a most vague and gratuitous hypothesis.

"Is it fair to argue from our ignorance? Because we do not know the precise manner in which a phenomenon occurs, shall we boldly assert the existence of new agencies in nature? Why by such logic we may multiply new principles almost *ad infinitum.* For instance, the nature of the diamond is perfectly well known; but can the chemist work backwards and form diamond from carbonic acid gas? Can he, in short, form any of those beautiful gems found in the mineral kingdom; such as topaz, ruby, etc.? He cannot. Then will it be urged that these inimitable productions of nature were formed by some force superadded to matter? Will some peculiar principle analogous to that termed *vital* be introduced to account for their formation? If so, let us at once go back to our exploded philosophy. Let us believe again that nature abhors a vacuum; and that this is a very satisfactory reason for the rise of liquids in exhausted receivers. Let us believe that the splendid researches of modern geologists amount to nothing; and that the impressions of organized beings, found in rocks, are in truth not those of beings once alive, but the productions of the *Vis Plastica* vainly laborring in the bowels of the earth, to evolve the forms of life. In short, let us shut at once the book of Induction, and open that of Hypothesis; which, though it really teaches us nothing, yet gives a pleasing play to the imagination, and gratifies our indolence by relieving us of all farther trouble.

"But it is asked, how comes it that tissues so various in structure, and of such diverse chemical constitution,—how comes it, such various secretions are formed in various localities, and all from one common substance, the arterial blood? It is answered, that these different formations are owing to the different circumstances under which the blood reacts with the solid molecules.

"From what has been said, it is plain, that to account for the formation of organic compounds, we have no good reason for saying that they are not solely produced by the operations of matter upon itself. The chemist it is true, cannot form albumen or fibrine from the elements of those substances, and for a very plain reason,—he cannot command the circumstances under which they were originally formed, and which were absolutely necessary to their formation."

The reasoning on which this rests is acute and ingenious, but there are few, we apprehend, who will not be rather startled at the conclusion, that the vital principle is a vagary, to be placed in the same category with the exploded hypothesis of phlogiston, and nature's abhorrence of a vacuum. This is going a step beyond Liebig. It is claiming for chemistry more than the chemists themselves arrogate for it. But if Professor Harrison is disposed to attribute too much to the plastic powers of chemical attraction, there are other physiologists who refuse it any agency in the evolution of vital phenomena. Truth, it is probable, lies between the extremes, in this case, as in so many others. These bold speculations of our author, we feel assured, will lead to good results by promoting inquiry.

Although Dr. Harrison styles his work an Essay on the

*Nervous* System, it embraces several other tracts on physiological subjects. It is from one of these—that on *life*—that we have made the foregoing extracts. Having, in the body of his work, treated of the "transmission of the nervous influence," of the "nature of the change undergone," of the "principles of natural philosophy and chemistry," of the "manner in which the change is transmitted," of "innervation," of "certain effects produced by the natural action of the nutritive fluids and solids," of "adynamia," of "hyperdynamia, or irritation," and of "ataxia," he goes on to explain certain phenomena upon the principles laid down under the foregoing heads. Some of these phenomena are the following; viz: Sensible contractility; contractility of the arteries; circulation of the capillaries; the pulse; sleep of plants; movements of the sensitivæ; ciliary motion; the motions of the Hedysarum gyrans, and of the heart; muscular motion; animal electricity; effects of dividing the pneumogastric nerves; and the function of the spleen. This accomplished, the author proceeds to make an application of all to pathology. The reader must be struck with the extent and variety of the bill of fare. The topics marshalled under a common head appear diverse enough, but they are shown to have among them points of resemblance which justify the grouping.

In the Appendix, which constitutes nearly half the volume, the author examines the "Electric theory of nervous action," and devotes many pages to the subject of life. It is in the latter chapter that we have been most interested, as in this, we think it is, that the logical powers of the author are exhibited to the greatest advantage. It closes with the following words, with which, reluctantly, we close our imperfect notice of this interesting work:

"Of a 'vital principle,' however, we can frame no more distinct notions than we can of phlogiston. As soon as we separate these principles from the substances which manifests the phenomena, they are words and nothing more.

"A metal, after being burned loses its 'phlogiston;' organic matter, after undergoing certain changes, loses its vital principle;'—the calx, burned with charcoal, is reduced to the metalic state, having regained its phlogiston; organic matter, being digested, becomes a part of the living being, having regained its 'vital principle.' The parallel is perfect.

"Another obstacle to the overthrow of long received opinions is our fondness for whatever is mysterious. To some minds, the nakedness of truth is appalling;—the roseate twilight of poetry being far more charming and attractive. It gladdens the imagination to occupy itself with what we deem the judgment cannot reach, and we shall scarcely take the trouble to fathom a subject which is particularly pleasing to the imagination. Superstition, soon, usurps the throne of reason; and questions, in themselves, perfectly innocent,—questions, that it is the duty of philosophy to propound, and, if it can, to answer,—come thus to be regarded with a holy horror. But this factious mystery, with which we enshroud natural objects, is the bane of science; whose object it is to lay bare the train of phenomena in their natural order of sequence. It, besides, diverts the mind from the perception of that great, that eternal mystery, which lies at the bottom of all things. 'All is wonderful, or nothing is.' The existence of a grain of sand with its powers and capabilities, is as incomprehensible and as wonderful as the existence of a star. He, who recognises this great truth, will smile at all human efforts to aggrandize what is already infinite, or to render more sacred the awful mystery of the universe. If we must have mystery, let us seek it there—in "that which lies at the bottom of appearance,' and remember always, that, *boni viri nullam oportet causam esse, præter veritatem.*" Y.

